# Supplementary material for: Assessing Phenotypic and Genotypic Resistance to Flumethrin in Varroa destructor Populations in Muğla, Türkiye
Source: Insects. 2025 May 22;16(6):548. doi: 10.3390/insects16060548 (PMC12193500; doi:10.3390/insects16060548)
Supplement: Supplementary file 1 [file insects-16-00548-s001.zip › insects-3557405-supplementary.pdf]

Assessing Phenotypic and Genotypic Resistance to Flumethrin in *Varroa destructor* Populations in Muğla, Türkiye

Ali Sorucu [1,2,*], Bekir Çöl [3,4*], Esra Dibek [4,5] and Anara Babayeva [4,6]

[1] Department of Pharmacology and Toxicology, Faculty of Milas Veterinary Medicine, Muğla Sitki Koçman University, Muğla, 48200 Türkiye
[2] Beekeeping and Silkworm Research and Application Centre, Muğla Sıtkı Koçman University,
Muğla 48200, Türkiye
[3] Department of Biology, Faculty of Science, Muğla Sitki Koçman University, Muğla 48100, Türkiye
[4] Biotechnology Research Centre, Muğla Sitki Koçman University, Muğla 48100, Türkiye; esradibek@mu.edu.tr (E.D.); anara.babazade@gmail.com (A.B.)
[5] Department of Pharmacy Services, Köyceğiz Vocational School of Health Services, Muğla Sitki Koçman University, Muğla 48800, Türkiye
[6] Biology Program, Graduate School of Natural and Applied Sciences, Muğla Sitki Koçman University,
Muğla 48000, Türkiye
* Correspondence: alisorucu@mu.edu.tr, bcol@mu.edu.tr.

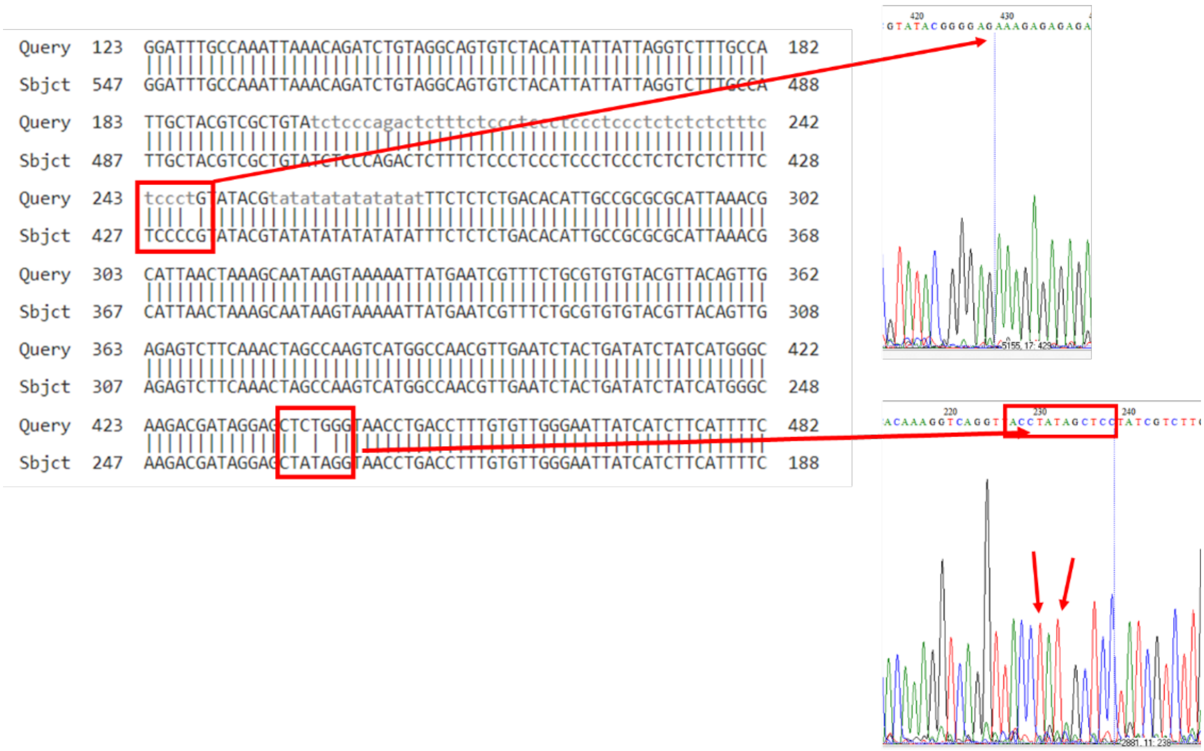

Figure S1 Bioinformatic analysis of the sequence result of sample V1

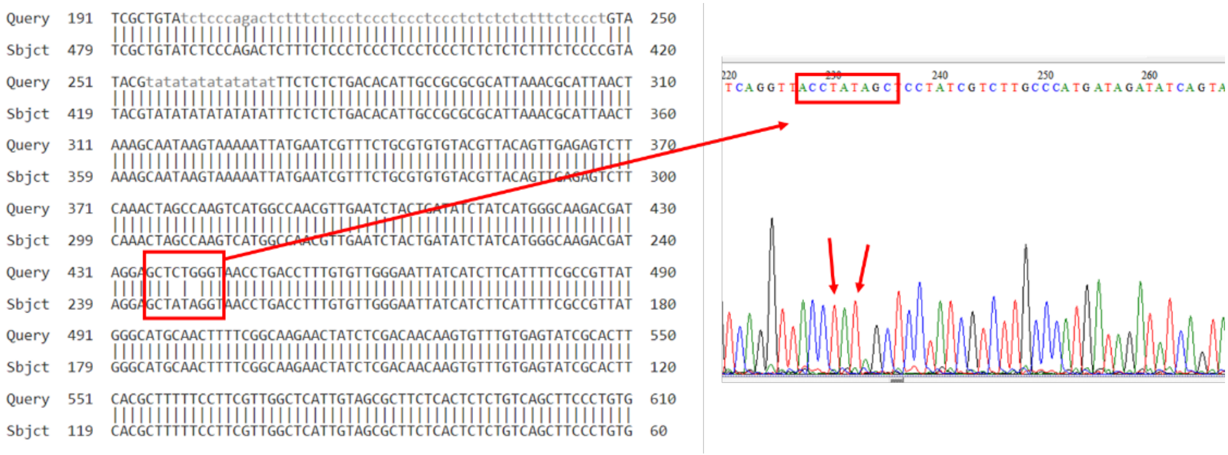

Figure S2. Bioinformatic analysis of the sequence result of sample V2

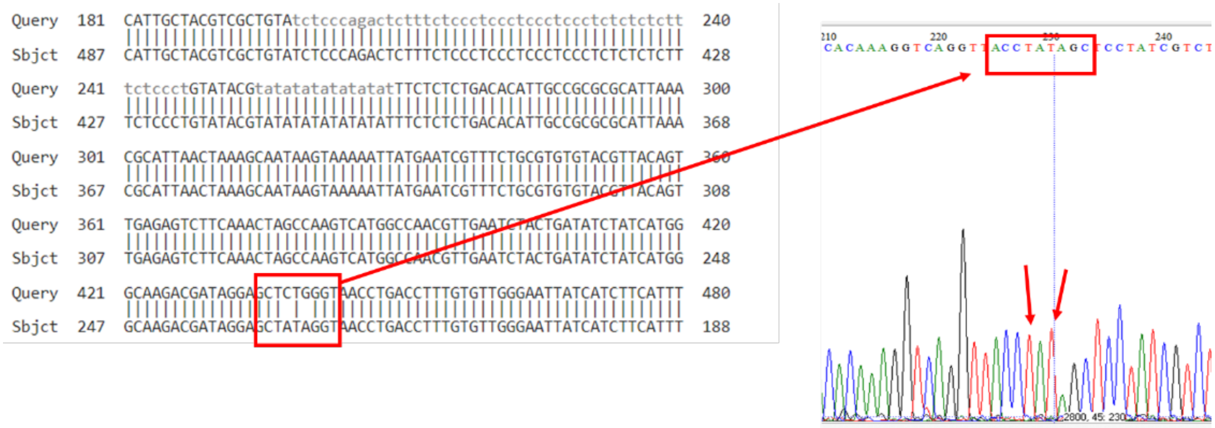

Figure S3 Bioinformatic analysis of the sequence result of sample V4

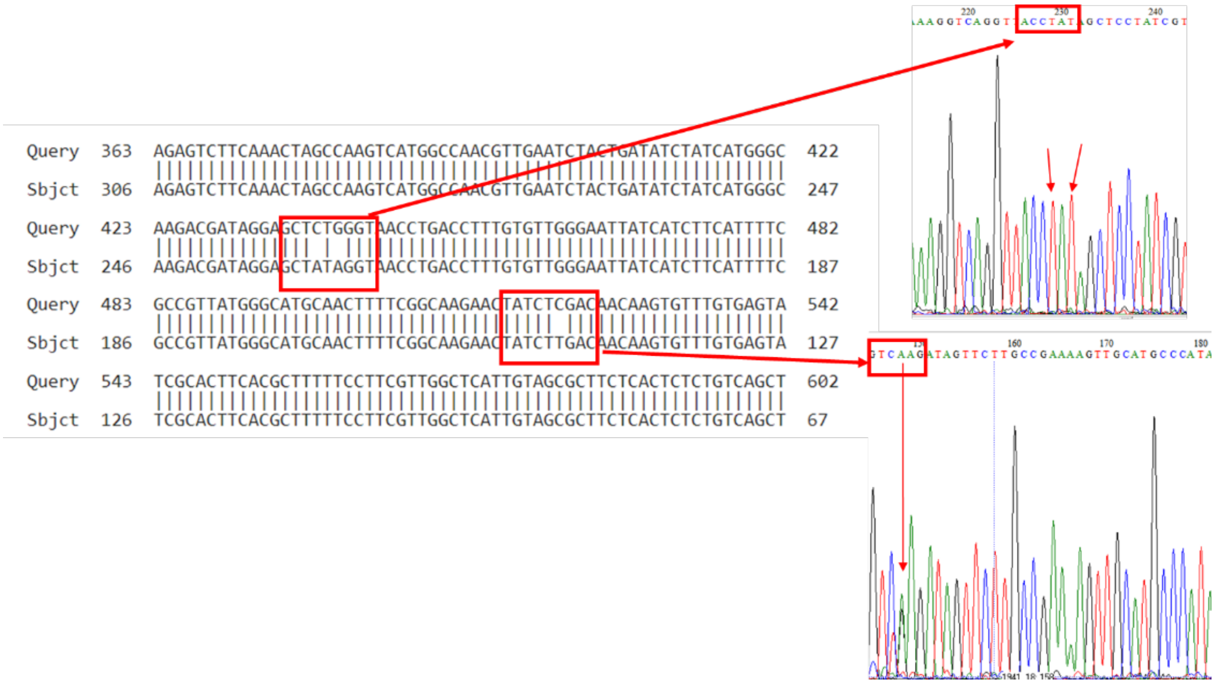

Figure S4 Bioinformatic analysis of the sequence result of sample V5

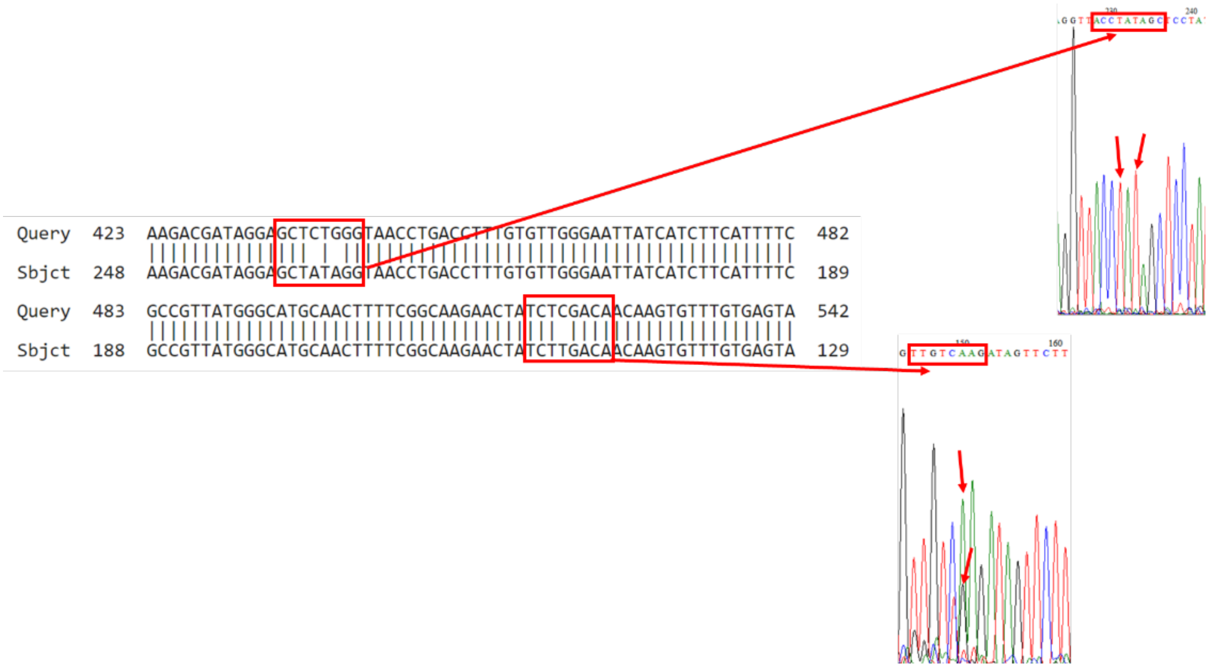

Figure S5 Bioinformatic analysis of the sequence result of sample V7

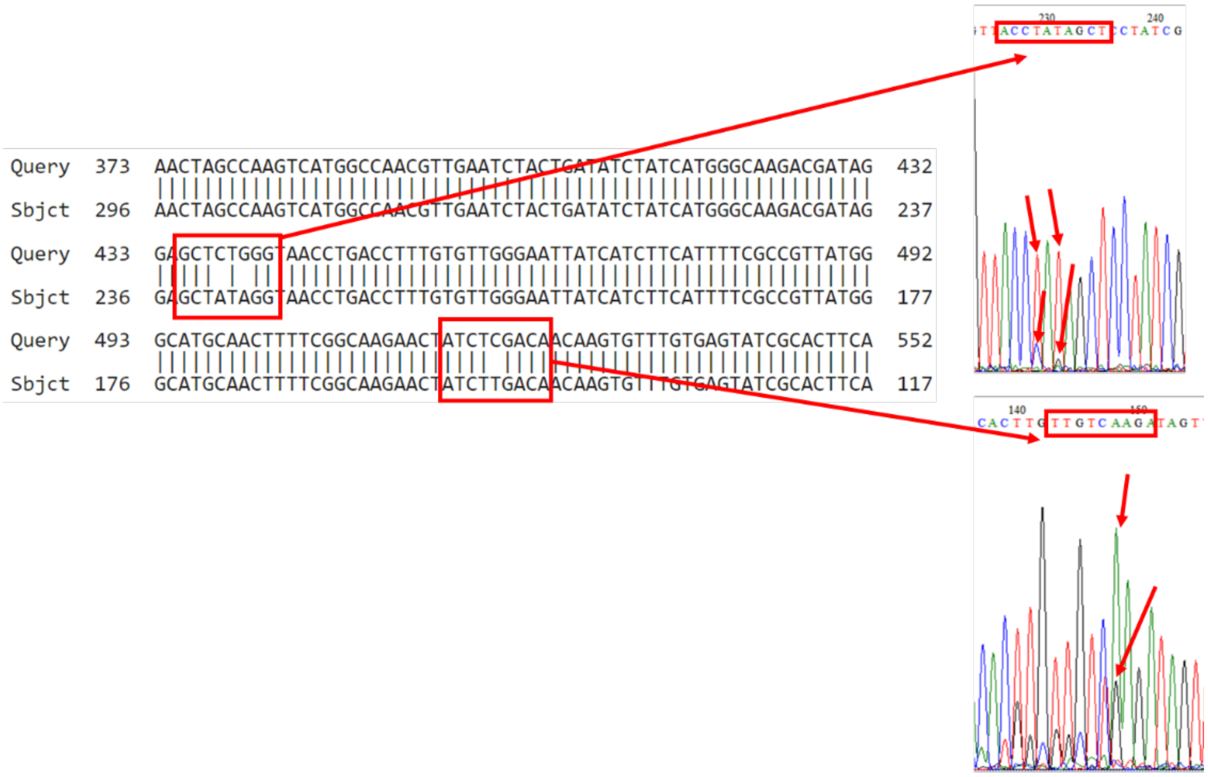

Figure S6 Bioinformatic analysis of the sequence result of sample V9

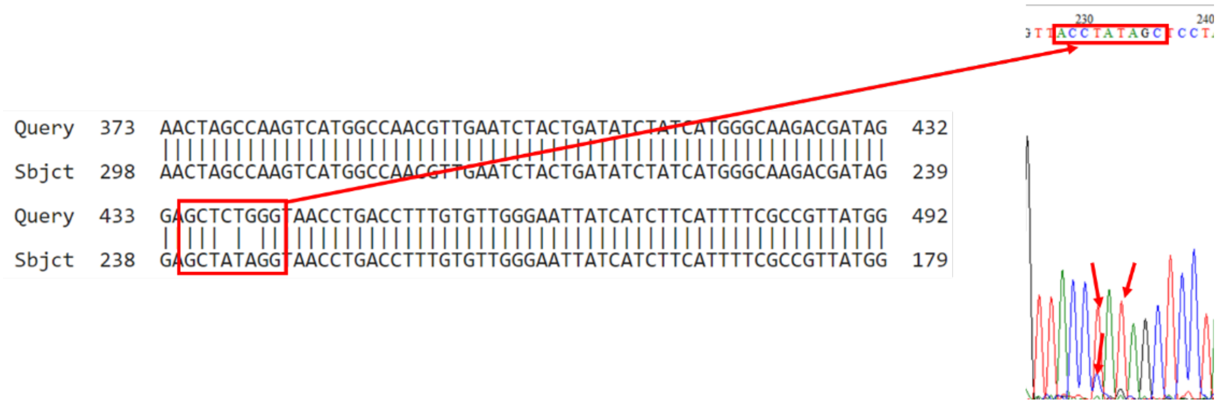

Figure S7 Bioinformatic analysis of the sequence result of sample V10

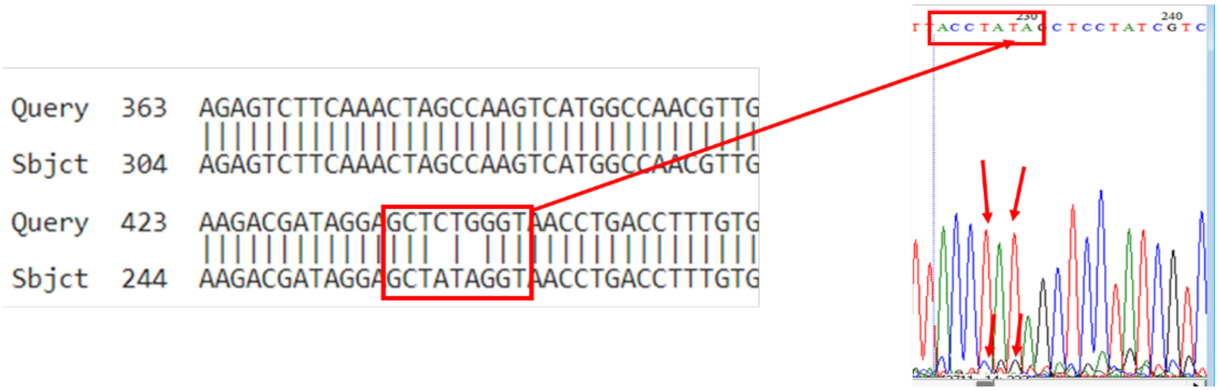

Figure S8 Bioinformatic analysis of the sequence result of sample V20

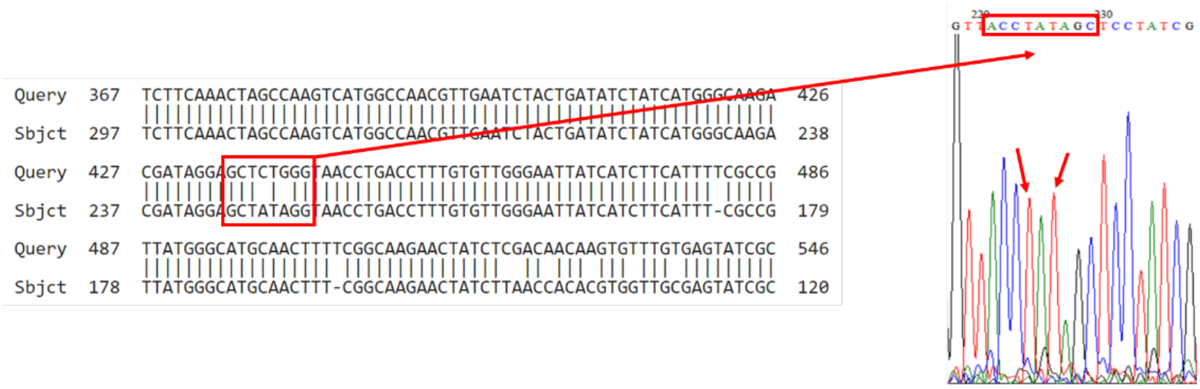

Figure S9 Bioinformatic analysis of the sequence result of sample V22
